# The effect of a large proximal haemodialysis arterio-venous fistula on weaning off cardiopulmonary bypass: case report

**DOI:** 10.1186/1749-8090-3-44

**Published:** 2008-07-09

**Authors:** Brian Nyawo, Amit Pawale, Leena Pardeshi, David Talbot, Jonathan Forty

**Affiliations:** 1 Department of Cardiothoracic Surgery, The Freeman Hospital, Newcastle Upon Tyne, NE7 7DN, UK; 2 Department of Cardiac Anaesthesia, The Freeman Hospital, Newcastle Upon Tyne, NE7 7DN, UK; 3 Department of Renal/Liver Transplant Unit, The Freeman Hospital, Newcastle Upon Tyne, NE7 7DN, UK

## Abstract

An increasing number of renal dialysis-dependent patients with Arterio-Venous fistulae are undergoing cardiac surgery.

The fistula has important effects on systemic hemodynamics in dialysis patients. The flow is significantly and positively related to cardiac output and cardiac index, and inversely related to pulmonary vascular resistance.

Few problems are encountered on cardiopulmonary bypass despite left to right shunting of blood. We present an unusual case in which a large brachial Arterio-Venous fistula with large collaterals prevented weaning off cardiopulmonary bypass.

## Introduction

Patients with chronic renal failure have a high prevalence of coronary artery disease and an increasing number are undergoing coronary artery bypass grafting. A-V fistula decreases mean systemic arterial pressure, effective systemic blood flow, total and pulmonary peripheral resistances, whereas it increases heart rate, total cardiac output, stroke volume, left atrial pressure, pulmonary arterial pressure [1,2]. In patients without cardiac failure, CO and CI are significantly higher in patients with elbow/upperarm access compared to patients with forearm access types. However, only a small percentage of patients with elbow/upperarm fistulae develop high-output cardiac failure.

In these patients few problems are encountered on cardiopulmonary bypass despite left to right shunting of blood. We present an unusual case in which a large A-V fistula with large collaterals prevented weaning off cardiopulmonary bypass.

## Case report

A 77 yr old gentleman was admitted with an acute coronary syndrome with modest troponin rise. He was known to have aortic stenosis and coronary artery disease and the aortic gradient had accelerated by 50 mmHg over a 6 month period to 80 mmHg with good systolic but poor diastolic function. Valve area was calculated at 0.7 cm^2 ^and coronary angiogram showed significant two vessel disease involving the LAD and circumflex territory.

He had End Stage Renal Disease for six years and was on chronic Haemodialysis via a surgical brachiocephalic Arterio-Venous fistula three times a week. The fistula was capable of flows averaging 400 ml/min during haemodialysis.

Prior to surgery he became unstable and required an IABP necessitating urgent mechanical aortic valve and myocardial revascularization procedures.

He had an uneventful anaesthetic induction and institution of cardiopulmonary bypass. During bypass it was noted that he required large doses of vasoconstrictors (pitressin infusion) to maintain perfusion pressure. He underwent a successful aortic valve replacement with a size 21 Tophat mechanical valve, reversed saphenous vein graft to obtuse marginal of circumflex and LIMA to LAD with a crossclamp time of 70 minutes.

Detailed haemodynamic monitoring was performed from induction and cardiopulmonary bypass as depicted in figure.

On attempting to wean him off bypass his diastolic pressure was very low which severely compromised coronary perfusion pressure and distended the Right ventricle. Three attempts were made to wean him off by-pass but all failed.

## A-V access compression

Temporary closure of the A-V access by a sphygmomanometer allowed successful weaning off bypass. The corresponding Pulmonary artery pressure and Cardiac Output values were compared before and after the A-V occlusion. Occlusion resulted in a transient decrease in cardiac output (from 11.4 ± 0.6 to 5.3 ± 0.5 l/min, *P *= 0.01) and systolic Pulmonary artery pressure (from 80.2 ± 3.8 to 34.6 ± 2.8 mmHg, *P *< 0.028).

**Figure 1:**
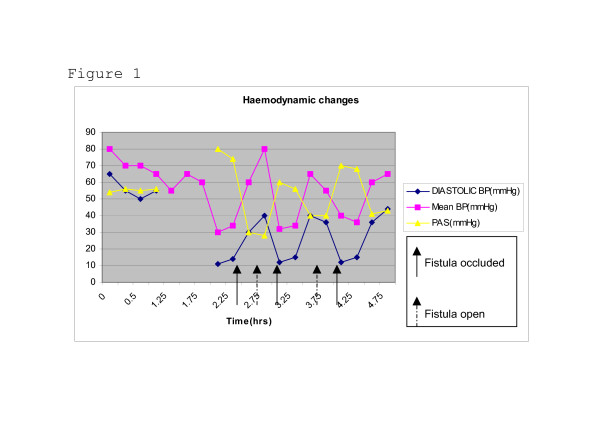
Haemodynamic changes.

**Figure 2:**
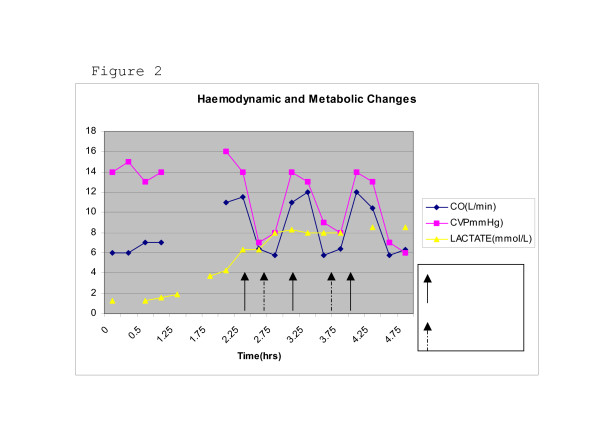
Haemodynamic and metabolic changes.

Similar flow and pressure measurements were obtained on three separate occasions of closure and release of compression. Eventually the fistula was explored and tied off together with 3 large collateral vessels. Flow characteristics at baseline and with arterio-venous fistula occluded are shown in Figure 1 &2. The patient was successfully weaned off bypass on IABP, NO and minimal inotropic support and was transferred to the ICU in a stable condition for further monitoring and care.

## Discussion

The haemodynamic effects of arterio-venous fistulae are well known [1-3] but the effects of this on cardiopulmonary bypass have not been described before. Short- and long-term [4,5] experiments have shown that AV fistulas cause an increase in CO due to enhanced venous return to the heart and subsequently exaggerated pulmonary blood flow. This hyperkinetic circulation can lead to cardiac failure or cardiac remodelling (compensatory hypertrophy of cardiomyocytes). Individual cases have been reported [6,7] where heart failure responded to closure or banding of the native AV fistula, but this is a very rare event.

In practice most patients have distal radial-cephalic fistulae where the flow is lower than brachio-cephalic fistulae and these are successfully weaned off bypass without problems. However, as in this case proximal fistulas with extremely high flow rates can cause problems in combination with the effects of surgery and cardiopulmonary bypass on normal physiology including activation of complement and other plasma protein systems. This leads to low SVR and PVR which compounds the affect of the shunt. In this case the presence of three large collaterals linked to the fistula may have further augmented A-V access flow. These multiple interacting factors were clearly demonstrated and reproducible on temporary occlusion of the fistula and made weaning off cardiopulmonary bypass virtually impossible because of extremely low diastolic pressures leading to heart failure.

Inadequate tissue perfusion and oxygenation was manifested by increased lactate production. The fistula may have contributed to this but other factors may have been responsible.

This case serves to highlight a potential problem of cardiopulmonary bypass in patients with chronic large proximal A-V fistulae which have developed collaterals. In such cases it may be wise to measure fistula flow prior to surgery and place an uninflated sphygmomanometer in anticipation of temporary occlusion.
